# Clinical presentation, management, and research progress of adrenal schwannoma

**DOI:** 10.3389/fsurg.2022.931998

**Published:** 2022-07-26

**Authors:** Shenghan Xu, Ying Yu, Yajuan Zhang, Yong Wen, Wei Li, Tao Huang, Bangwei Che, Wenjun Zhang, Jinjuan Zhang, Kaifa Tang

**Affiliations:** ^1^ Department of Urology, The Affiliated Hospital of Guizhou Medical University, Guiyang, China; ^2^ Department of Pathology, The Affiliated Hospital of Guizhou Medical University, Guiyang, China; ^3^ Department of Imaging, The Affiliated Hospital of Guizhou Medical University, Guiyang China; ^4^ Department of Imaging, The Third People's Hospital of Guiyang, Guiyang, China; ^5^ Basic Medical College of Guizhou Medical University, Guiyang, China

**Keywords:** adrenal gland, schwannoma, adrenalectomy, computed tomography, immunohistochemical staining

## Abstract

**Objective:**

This study shares our experience in managing adrenal schwannoma (AS).

**Methods:**

The clinical data of eight patients with AS in our hospital from April 2007 to April 2022 were analyzed retrospectively.

**Results:**

A total of 1309 patients with adrenal lesions were treated in the affiliated hospital of Guizhou Medical University for 15 years, of which only 8 cases were diagnosed as AS, accounting for 0.61%. Among the eight patients with AS, there were five females and three males, with an average age of 48.63 ± 12.05 years, and the average maximum diameter of the tumor was 6.96 ± 1.83 cm. All patients underwent adrenalectomy and were pathologically diagnosed as AS after the operation. The average follow-up time of eight patients with AS was 60.13 ± 22.33 months, and there was no recurrence or metastasis.

**Conclusion:**

The retroperitoneum is an uncommon site for schwannoma tumors, and among adrenal incidentalomas, the schwannoma is rare. The disease lacks specific clinical and imaging features, but correct diagnosis before the pathological examination is very important for clinical management and surgical decision. When imaging examination indicates a slow-growing retroperitoneal mass, schwannoma should be considered. Surgical resection is the main treatment. Pathology is the gold standard for diagnosis. Most of the tumors are benign and have a good prognosis. There is a risk of recurrence after the operation, and it should be monitored actively.

## Introduction

Adrenal schwannoma (AS) is an uncommon and slow-growing incidental tumor that usually originates from Schwann cells in the nerve sheath of the adrenal gland, mostly benign tumors and a few malignant tumors (1). Schwannomas usually occur in the skull, head, neck, and limbs but rarely in the retroperitoneal space. Most AS are found accidentally by physical examination. Most patients have no obvious clinical symptoms and may have symptoms such as abdominal pain and low back pain due to compression (2, 3). Patients with AS lack specific signs or imaging findings, which can easily lead to misdiagnosis before surgery. The prognosis is good after surgical resection of the tumor, but it is easy to relapse after the operation and needs close follow-up (4).

Due to the low incidence of AS, only a few case reports and small case series have been published so far, and the total number of AS cases is less than 200 (5). From April 2007 to April 2022, the affiliated hospital of Guizhou Medical University treated 1309 patients who underwent surgical resection of adrenal lesions, of which only eight were diagnosed as AS, accounting for only 0.61%. Therefore, a comprehensive understanding of the clinical features, diagnosis, treatment, and prognosis of AS is of great significance. We collected the data of eight patients with AS confirmed by pathology and analyzed the characteristics of the diagnosis and treatment of the disease in combination with the literature.

## Materials and methods

This study was approved by the Ethics Committee of the affiliated hospital of Guizhou Medical University. From April 2007 to April 2022, eight patients with AS underwent surgery in the Department of Urology, Affiliated Hospital of Guizhou Medical University. We retrospectively collected the clinical data, imaging features, endocrine indicators, treatment measures, pathological diagnosis, and follow-up data of these patients. All patients underwent computed tomography (CT). The following contents were reviewed by an imaging expert (Professor Yong Wen): tumor location, maximum diameter, shape, necrosis, cystic, calcification, interval, signal strength, and enhancement pattern, and finally the preoperative diagnosis was confirmed. During the initial examination at the hospital, all patients had normal blood pressure and were confirmed to be nonfunctional adrenal tumors, which were followed by laparoscopic adrenalectomy. All pathological sections were retrospectively analyzed by a pathologist (Professor Yajuan Zhang) with professional knowledge of urinary tumors. The routine follow-up plan was once every 6 months in the first year and once a year after the second year.

## Results

### Clinical information

Table 1 summarizes the basic demographic characteristics and clinical manifestations of eight patients with AS. Among them, five cases were females and three cases were males. Their average age was 48.63 ± 12.05 years old. Among them, five cases had low back discomfort, one case had epigastric pain, and the other two cases were accidentally found by physical examination. Before the operation, blood pressure, endocrine function, and serum potassium levels were normal in all patients.

**Table 1:** Demographic and clinical characteristics of eight patients with adrenal schwannoma.

Patient characteristics	*n* (%)
Mean age at onset (range) (years)	48.63 ± 12.05(24∼63)
Sex	
Female	3(37.5)
Male	5(62.5)
Tumor site	
Left	4(50)
Right	4(50)
Average tumor diameter (range) (cm)	6.96 ± 1.83 (4.4∼9.7)
Reason for visit	
Asymptomatic (physical examination)	2(25)
Lower back pain	5(62.5)
Epigastric pain	1(12.5)
Imaging features	
Necrotic	7(87.5)
Calcification	5(62.5)
Cystic degeneration	7(87.5)
Interval	3(37.5)
Pathological features	
Type Antoni A is dominant	0(0)
Type Antoni B is dominant	0(0)
Mixing of Antoni A and Antoni B	8(100)
Preoperative diagnosis	
Neurogenic tumor	3(37.5)
Pheochromocytoma	5(62.5)
Mode of operation	
Open surgery	6(75)
Laparoscopic surgery	2(25)
Prognosis	
Recurrence	0(0)
No recurrence	8(100)

### Imaging features

**Figure 1:**
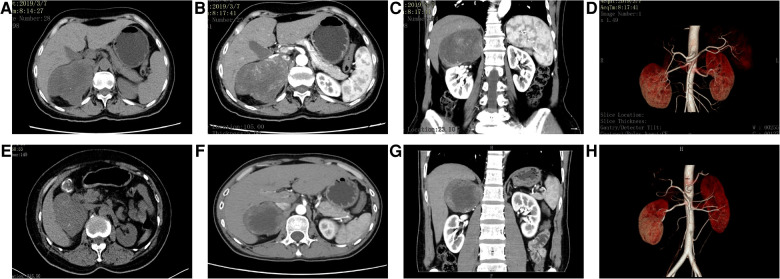
CT images of some patients. (**A–D**) Case 1: A mass-like mixed-density shadow was seen in the right adrenal area, and the boundary was still clear. The contrast-enhanced scan showed inhomogeneous progressive enhancement; the focus was mainly supplied by the small artery from the abdominal aorta, and the right renal artery was depressed. (**E–H**) Case 6: The mass density in the right adrenal area was slightly lower and the boundary was clear; the enhanced scan showed mild to moderate enhancement at the edge of the lesion, no enhancement in the central necrotic area, and compression changes in the liver and right kidney. The focus was mainly supplied by the right middle adrenal artery and inferior artery. Part of the blood vessels entered the mass, and the right renal artery moved downward under pressure.

Table 2 summarizes the CT findings of eight patients with AS. In our study, the average maximum diameter of adrenal tumors in eight patients was about 6.96 ± 1.83 cm. All tumors were round or quasi-round masses, which occurred in the unilateral adrenal gland. The tumor boundary was not clear in two cases (25%), intratumoral necrotic changes were observed in seven cases (87.5%), calcification was observed in five cases (62.5%), and cystic changes were observed in seven cases (87.5%), and septum was observed in three cases (37.5%). The CT images of some patients are shown in Figure 1. The unenhanced CT values of eight cases ranged from 30 to 43 Hounsfield units (HU) (mean 35.71 ± 4.56 HU), and the enhanced scans showed mild to moderate delayed enhancement, ranging from 34 to 63 HU (mean 48.05 ± 9.06 HU). No malignant features were found in all cases, such as invasion of adjacent tissues or distant metastasis. Preoperative CT was considered the neurogenic tumor in three cases (37.5%) and pheochromocytoma in five cases (62.5%).

**Table 2:** Clinical and CT features of eight patients with adrenal schwannoma.

Case number	Sex/Age	Symptoms	Location/Largest tumor diameter (cm)	Tumor edge morphology	Necrosis	Calcification	Cystic degeneration	Interval	CT attenuation (HU)				Preoperative imaging diagnosis
									Plain scan	Arterial phase	Venous phase	Delayed phase	
1	F/59	Right back pain	Right/9.7	Irregular	+	−	+	−	31	52	65	74	Neurogenic tumor
2	F/53	Epigastric pain	Right/7.8	Irregular	+	+	+	−	43	63	79	86	Neurogenic tumor
3	F/63	No clinical symptoms	Left/4.4	Rule	−	+	−	−	30	34	55	56	Neurogenic tumor
4	M/60	Left lower back pain	Left/6.2	Rule	+	+	+	+	32	37	61	63	Pheochromocytoma
5	F/24	No clinical symptoms	Left/5.6	Rule	+	−	+	+	36	50	63	82	Pheochromocytoma
6	M/44	Right back pain	Right/7.3	Rule	+	−	+	+	34	44	57	67	Pheochromocytoma
7	F/45	Left lower back pain	Left/6.8	Rule	+	+	+	−	40	56	63	70	Pheochromocytoma
8	M/41	Right back pain	Right/6.7	Rule	+	+	+	−	38	52	60	64	Pheochromocytoma

CT, computed tomography; HU, Hounsfield unit.

### Management

Adrenalectomy was performed in eight patients with AS, of which two patients (cases 3 and 5) underwent laparoscopic surgery and the other six patients underwent open surgery. In case 1, we once thought it was a malignant tumor during the operation. After opening the peritoneum and entering the abdominal cavity, we found that the adhesion between the tumor and the liver, intestinal tract, and tissue around the renal vein was serious, and the anatomical structure was not clear, which caused the rupture of the renal vein when the adhesion of the renal vein was separated. Then, the renal vein was repaired, and finally, the adrenal tumor and surrounding adhesive tissue were completely removed. All patients recovered well after the operation and were discharged smoothly.

### Histopathology

**Figure 2:**
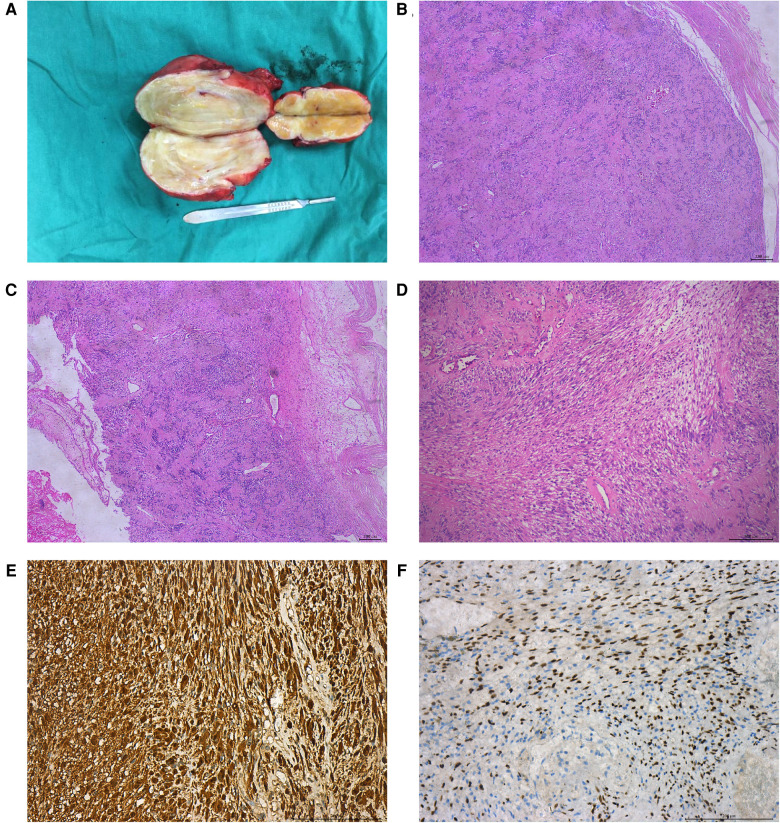
Pathological features of patients with benign AS. The gross specimen of the tumor was a gray-white round-like mass (**A**); HE staining showed that the tumor was alternately composed of the Antoni A area and the Antoni B area, showing verrucous body (**B**); mucous stroma (**C**); and transparency (**D**); immunohistochemical staining showed that S-100 protein (**E**) and SOX-10 protein (**F**) were positive.

The pathological features of eight patients with AS are shown in Table 3. Most of the gross specimens of the tumor are round-like solid masses (Figure 2A), and the section is usually composed of hard gray-white tissue. Microscopic examination showed that the capsule of AS was intact in eight cases, which was composed of alternating high cell areas (Antonia-type tissue) and low cell areas (Antoni B-type tissue) (Figure 2B). Verocay bodies were seen in four cases (Figure 2B), myxoidstroma in four cases (Figure 2C), and hyalinization in three cases (Figure 2D). No necrotic changes were found in all cases. Immunohistochemical staining showed that S-100 protein (Figure 2E) was diffusely expressed in tumor cells in all cases and SOX-10 protein (Figure 2F) was positive in some cases. These findings support the diagnosis of benign AS.

**Table 3:** Pathological features of eight patients with adrenal schwannoma.

Case	Microscopic characteristics of tumor cells							Immunohistochemistry					Follow-up (months)
	Capsule	Antoni A/B	Verocay body	Microcystic	Necrosis	Myxoid stroma	Hyalinization	S-100	SOX-10	Vim	CD34	Ki-67	
1	+	+/+	+	+	−	+	+	+	+	+	−	2%+	30/Survive
2	+	+/+	+	−	−	−	−	+	+	−	−	1%+	36/Survive
3	+	+/+	−	−	−	−	−	+	NA	NA	−	2%+	48/Survive
4	+	+/+	+	−	−	+	+	+	+	+	−	5%+	50/Survive
5	+	+/+	−	−	−	−	+	+	+	+	−	1%+	90/Survive
6	+	+/+	+	−	−	−	−	+	NA	+	−	3%+	95/Survive
7	+	+/+	−	−	−	−	−	+	+	−	−	1%+	60/Survive
8	+	+/+	−	−	−	+	+	+	+	+	−	2%+	72/Survive

NA, not available.

### Follow-up

All patients with AS were followed up actively after surgery. The average follow-up time was 60.13 ± 22.33 months, no recurrence or metastasis occurred, and all patients survived.

## Discussion

Schwannoma originates from Schwann cells of the peripheral nerve sheath, so it is also known as a Schwann cell tumor. It was first reported by Verocay in 1908 and divided into two subtypes by Antoni in 1920 (6). Neurilemmoma usually occurs on the flexor side of the head, neck, and extremities, and it is rare in retroperitoneal cases. Schwannomas account for about 0.5%–1.2% of retroperitoneal benign tumors, while ASs account for about 0.7%–2.7% of all schwannomas (7). At present, most scholars believe that AS originates from the adrenal medulla innervation of the retroperitoneal nerve tissue and has nothing to do with the adrenal tissue itself (8). Just because the tumor occurs in the retroperitoneal adrenal region, we habitually call it “AS” according to its location. In fact, it should be called para-adrenal neurilemmoma (1).

AS can occur at any age, but it is mainly seen in female patients between 40 and 60 years old (9). The demographic characteristics of our case group are consistent with those reported in the literature. Most of the tumors are benign, but there are also reports of malignant or metastatic AS (9). The vast majority of retroperitoneal schwannomas are located in the paraspinal space or prevertebral region. Because there is a lot of space in the retroperitoneum, schwannomas need to reach a larger size before symptoms appear, so they hardly cause any symptoms in the early stage. Most patients were observed for physical examination or other reasons, and a few patients had symptoms such as lumbar or abdominal pain or upper abdominal distension (10). The diagnosis of AS during pregnancy has been reported in the literature. Retroperitoneal schwannoma can mimic the characteristics of female genital masses during pregnancy, such as adnexal mass, ectopic pregnancy, and so on (11). However, because we cannot use contrast media during pregnancy, this will make our diagnosis more difficult. The continuous growth of retroperitoneal schwannoma oppresses the fetus and its surrounding organs, which increases the risk of pregnancy. The correct early diagnosis of retroperitoneal schwannoma during pregnancy can save the lives of both the fetus and the mother to a certain extent (12). General laboratory tests such as serum electrolytes, renin, aldosterone, catecholamine, and cortisol are mostly within the normal range, and most adrenal tumors are nonfunctional (unable to produce steroids or catecholamines) (13). Therefore, it shows the characteristics of nonsecretion, is asymptomatic, and is often diagnosed by accident. Referring to the previous literature, the size of AS ranges from 0.6 to 14 cm, and the average maximum diameter of the tumor is about 5.5 cm (7, 14). In our study, the average maximum diameter of the tumor was about 6.96 ± 1.83 cm, which is larger than that reported in the literature, which may be related to the late arrival time of the patient at our institution. AS has no biological evidence to prove the correlation between tumor size and malignant biological potential (14). In this case group, some patients with tumors were found late due to asymptomatic, delayed treatment, resulting in large tumors, making the operation more complex and difficult.

It is difficult to diagnose AS before the operation, and the final diagnosis mainly depends on the pathological diagnosis. AS will lead to secondary degeneration due to insufficient blood supply of the tumor. The larger the tumor, the more obvious the rearrangement, such as cyst formation, calcification, bleeding, transparency, and so on (7). The histopathology of schwannoma consists of the Antoni A region and the Antoni B region (15). The Antoni A region is closely arranged by Schwann cells, and the nucleus is arranged in a palisade shape, which makes it difficult to be cystic (15). In the Antoni B region, Schwann cells are arranged in a loose reticular structure, which often occurs after cystic degeneration or hemorrhage (15). Most of the Antoni A region and the Antoni B region coexist in the same tumor, and the two structures are intertwined. The Antoni A region is mostly distributed in the periphery of the tumor, and the Antoni B region is mostly located in the center of the tumor (16). The proportion, distribution, and arrangement of the Antoni A and Antoni B areas in tumors are different, and there will be different CT manifestations, so it is very difficult to make a definite diagnosis before operation. On CT, most of the Antoni A area showed high density, which could be enhanced after enhancement, while the plain scan density of the Antoni B area was lower, and the enhancement was not obvious after enhancement (17). Most of the gross specimens of benign AS are solid, well-defined brown-yellow round masses (18). The cellular variation of AS seems to be more common than that of schwannomas in the superficial soft tissue of the trunk. Large AS can be seen under a microscope with bleeding, calcification, or cystic changes. This corresponds to the degenerative changes observed in imaging (19). Like other schwannomas, AS may have the following histological features, such as intact capsule, Antoni A and B areas, verocay body, hyalinization, myxoid stroma, and foam cells. The immunohistochemical features of benign AS have the following commonalities: most are positive for S-100 or SOX-10, but negative for keratin, desmin, actin, CD34, and CD117 (5, 20). The positive expression of Ki-67 in eight patients with AS was less than 5%, and their low proliferation index suggested that they had the biological nature of laziness. Combined with the analysis of literature and clinical data (18, 21–23), we think that the following characteristics play a role in the diagnosis of AS: (1) It is common in female patients, and the clinical symptoms are mild or even without any clinical symptoms in the early stages, and the excessive volume of the tumor may lead to the displacement of the surrounding organs in the late stage. (2) The tumor is a well-defined round or irregular mass, most of which are unilateral and large, generally >5.5 cm. (3) Under CT, most tumors showed cystic or hemorrhagic degeneration, delayed septal enhancement, and mild enhancement, showing inhomogeneous low-density lesions, while a few solid tumors showed uniform density and CT value similar to muscle signal intensity, which could be significantly enhanced. (4) Calcification is an important feature of retroperitoneal schwannoma. CT findings may be punctate, mottled, or curvilinear. (5) Cystic degeneration is the final stage of long-term growth and evolution of tumors, and most tumors may have cystic degeneration. (6) The “rabbit tail sign of distal adrenal gland” under CT may indicate AS. (7) On MRI scan, AS showed low signal intensity on T1W1 and inhomogeneous high signal intensity on T2W1. (8) Most of the tumors had no endocrine function. (9) Immunohistochemistry showed positive expression of S-100 or SOX-10. Considering that AS is relatively rare, there are few studies on AS at home and abroad, most of which are case reports. We need a larger clinical case review to support the above conclusions. In addition, adrenal tumors can also be percutaneously biopsied, but a biopsy may lead to tumor overflow, bleeding, and other adverse complications (24). This examination is applicable when the patient has lost the opportunity for surgery and needs a clear diagnosis to help with the next step of treatment. This examination is an invasive operation and may lead to missed diagnosis, so routine use is not recommended.

The preoperative imaging misdiagnosis rate for this group of patients is 62.5%. Such a high misdiagnosis rate is related to our small number of cases and the lack of specific imaging findings, which makes it very difficult to differentiate from other adrenal tumors before an operation. We comprehensively consult the literature and summarize some other adrenal tumors that are easy to be confused with schwannoma, which is helpful to improve the differential diagnosis between doctors and other diseases before the operation and reduce the overtreatment of patients caused by preoperative misdiagnosis, to formulate a reasonable treatment plan (25–28). AS is difficult to distinguish from the following diseases: (1) Adrenal nonfunctional adenoma: because the tumor has no obvious endocrine abnormality, it is often accidentally found that most of the tumors are round or quasi-round nodules with clear boundaries, and the T1WI tumors containing lipids show high signal intensity, while the inverse phase T1WI tumor signals are decreased. This kind of adenoma can be easily differentiated from neurilemmoma. However, the enhanced scan of adenomas lacking lipids is generally mild to moderate enhancement in the early stages, so it is difficult to distinguish them from schwannomas. (2) Adrenal pheochromocytoma: adrenal pheochromocytoma is a common tumor of the adrenal medulla, mostly in young adults, due to increased catecholamine secretion in patients’ blood, paroxysmal or persistent hypertension, and metabolic disorders. When the tumor was small, it showed a solid mass, and when it was larger, the center often showed cystic degeneration due to hemorrhage and necrosis. The solid part of the tumor was significantly enhanced, and a few lesions could have plaque calcification; T2WI showed obvious high signal intensity, while the contrast-enhanced scan showed rapid and significant enhancement. (3) Adrenal ganglioneuroma: a benign tumor that develops from the adrenal medulla. The tumor was round or irregular, with a capsule, and showed low density on CT, in which punctate calcification was common, and tumor enhancement was not obvious on the enhanced scan, but cystic degeneration, necrosis, and hemorrhage were rarely seen. T1WI showed uniform low signal intensity, while T2WI showed inhomogeneous high signal intensity, no obvious enhancement, and delayed enhancement in the early stages of the enhanced scan. (4) Adrenal neuroblastoma: mostly in children, CT showed a round cystic solid mass or irregular solid mass, uneven density, necrotic area and flaky calcification at the center, vascular entrapment, and vascular dilatation in or around the tumor. The typical MRI findings were calcification, necrosis, hemorrhage, and cystic degeneration. T2WI showed low signal intensity, and T2WI showed high signal intensity. (5) Adrenal cyst: it can occur at any age and can be divided into true cysts and pseudocysts. True cysts showed watery density with thin walls without enhancement; most of them were unilateral; a few of them had calcification; the thickness of the pseudocyst wall was uneven, and most of them were accompanied by calcification. With the accumulation of clinical experience and the improvement of science and technology, we have reason to believe that the preoperative misdiagnosis rate of AS will be greatly reduced.

Asian people are more likely to develop occasional adrenal tumors than those in Europe (27, 29). At present, the most effective way to treat AS is through surgical resection of the tumor. Because its clinical symptoms are not obvious and its volume is large when it is found, it is difficult to accurately judge whether the tumor is benign or malignant before operation (30). Most scholars advocate that the tumor and the surrounding adhesive tissue should be removed completely during the operation. The specific model of operation should be determined by the size of the tumor, its adjacent relationship with blood vessels and surrounding tissues, and whether there are signs of local invasion or not (30). With the development of laparoscopic technology, the technique of laparoscopic adrenal tumor resection is becoming more and more mature. Laparoscopic surgery has the advantages of less trauma and rapid recovery. Laparoscopic surgery has the advantages of less trauma and rapid recovery (30). There is a slight difference between Chinese and Western scholars in the surgical indications of incidental adrenal tumors. In China (31–33), most urologists recommend close monitoring for incidental adrenal tumors that are asymptomatic, nonfunctional, have no signs of malignancy, and whose tumor diameter is less than 3 cm. Surgical treatment is recommended for incidental adrenal tumors with endocrine function, malignant signs in imaging, or tumor diameter ≥3 cm. Laparoscopic surgery or Leonardo da Vinci robot surgery can be used for adrenal tumors with a diameter <6 cm or no local invasion. However, with the increase in the size of adrenal tumors, larger schwannomas are often surrounded by more blood vessels, which increases the difficulty and risk of operation. Open surgery is the first choice for adrenal tumors with tumor diameter ≥6 cm or local invasion. Most Chinese urologists prefer the retroperitoneal approach when performing laparoscopic adrenal surgery, and its advantage is that the retroperitoneal approach does not affect the intestine, minimizing the occurrence of intestinal obstruction and allowing rapid recovery of intestinal function after the operation. The retroperitoneal approach can quickly find the adrenal gland and renal hilum, shorten the operation time, and allow the patient to recover quickly after the operation. In the West, such as the National Institutes of Health/American Society of Endocrine Surgery (NIH/AAES) (34, 35), it is recommended that incidental adrenal tumors with tumor diameters of 4 cm, no malignant features, and no endocrine function should be monitored. Tumors ranging from 4 to 6 cm in diameter can be monitored or resected. Surgical treatment is recommended for tumors with rapid growth, uneven shape, irregular shape, necrosis, and infiltration of adjacent structures. Active surgical treatment is recommended for tumors with a tumor diameter ≥6 cm. Because of the large size of the tumor, it is often complicated with cystic degeneration and soft texture, so it should be squeezed gently when exposing the tumor to avoid tumor rupture. The operation process should abide by the “tumor-free principle.” Although AS usually does not invade neighboring organs, the tumor is too large and causes nearby structures to shift. It is worth noting that our case 1 is rich in blood vessels, with serious adhesion with surrounding tissue, partial loss of renal vein in free renal vein adhesion, and intraoperative blood loss of 3500 ml. After our active rescue and treatment, the patient was successfully discharged from the hospital. When the tumor is large or has severe adhesion with the surrounding tissue, attention should be paid to avoid damaging the renal artery and its branches, renal vein, and accessory renal artery when separating the tissue. In particular, the relationship between the tumor and the surrounding blood vessels should be clarified during the transabdominal approach, and the injury of arteries and veins should be avoided as far as possible. Tumor growth will cause organ and tissue compression and displacement, larger retroperitoneal tumor is more likely to cause bladder compression, resulting in a series of symptoms of stress urinary incontinence (36). Studies have shown that urinary incontinence can affect sexual function regardless of its nature (37, 38). Surgical resection of the tumor can reduce the symptoms of urinary incontinence and improve sexual function and quality of life (36–38).

The author believes that AS is easy to misdiagnose because of its rarity and lack of specific imaging findings. If it is difficult to rule out pheochromocytoma before the operation, full preoperative preparation is needed. To prevent excessive fluctuations in blood pressure during and after surgery, it is recommended to expand blood volume 7–10 days before the operation and prepare drugs such as α-blockers and β-blockers. Only 5%–10% of benign schwannomas recur after complete resection (39). However, if the tumor is not completely removed, 10%–54% of cases will have a local recurrence after the operation (40). Benign schwannoma has a good prognosis, but there is the possibility of recurrence and malignant transformation. Wilson et al. (11) recently reviewed and identified 121 reported ASs in the literature, of which the average size of the tumor was about 6.5 cm, about 50% of the reported cases had punctate calcification, and about 50% of the cases showed cystic/necrotic changes on imaging. In our case group, the incidence of tumor necrosis/cystic changes in imaging findings was 87.5%, which was much higher than the average level. Compared with schwannomas in other parts of the body, abdominal schwannomas are rare and need to be surgically resected completely. Although the imaging technology is very advanced, abdominal schwannomas lack imaging specificity, so preoperative diagnosis is still challenging. Gastrointestinal schwannomas are also rare and can be found in the stomach, small intestine, colon, or rectum. They are often found clinically because abdominal masses are found, but there are few symptoms, such as abdominal pain. Gastrointestinal schwannomas can easily lead to complications such as bleeding or intestinal obstruction (41). Gastrointestinal schwannomas not only have the typical histological features of schwannomas but also show surrounding lymphoid cells, which are difficult to distinguish from gastrointestinal stromal tumors on CT or MRI (42). Pancreatic schwannoma is a kind of slow-growing encapsulated benign tumor, and its occurrence is very rare (43). Pancreatic schwannoma most often involves the head of the pancreas, followed by the body of the pancreas, neck, tail, and uncinate process. Pancreatic neurilemmoma may be misdiagnosed as a pancreatic tumor before surgery, and doctors may remove pancreatic parenchyma, which is very disadvantageous to patients (44). Compared with schwannomas occurring in the gastrointestinal tract and pancreas, retroperitoneal schwannomas tend to be larger and more uneven in density (45). Due to the large space in the retroperitoneal cavity, most patients usually do not have any symptoms. Patients often have large tumors when they go to the hospital, which can mimic more primary adrenal tumors (46). The main purpose of this article is to comprehensively review the imaging findings of AS, which will be helpful in learning the diagnosis and differential diagnosis of preoperative schwannoma to guide the treatment plan and avoid unnecessary radical resection or resection of adjacent organs, to provide patients with optimal treatment (7, 8, 46).

**Figure 3:**
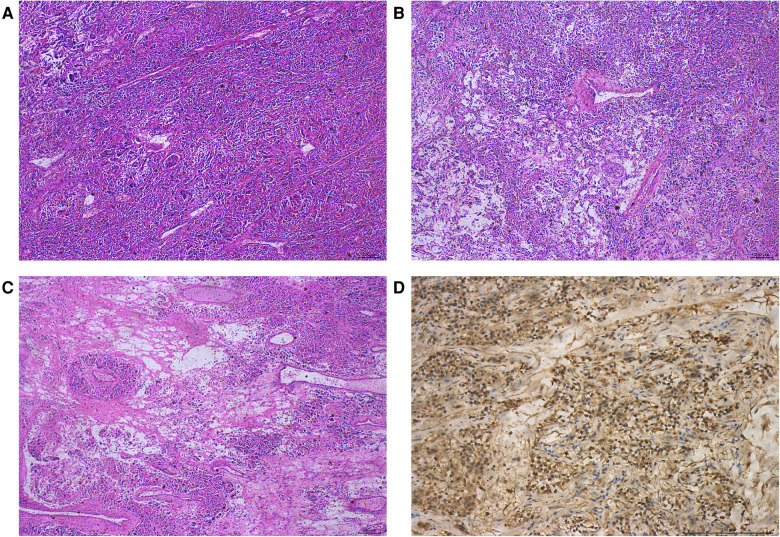
Pathological features of malignant AS. HE staining showed that the tumor was mainly in the Antonia area (**A**); transparent vessels and local myxoid degeneration (**B**); adenoid/microcapsule structure (**C**); and immunohistochemical staining showed positive expression of S-100 protein (**D**).

Jow et al. (47) reported the first case of malignant adrenal neurilemmoma in 1991. Since 1991, most of the cases of malignant AS have been reported sporadically in the form of case reports, which lack statistical support. Malignant neurilemmoma is most common in the extremities, followed by the trunk, head, and neck, and rarely in the adrenal gland. We found an interesting thing: most of the patients with malignant adrenal sheath tumors are children or adolescents, and their diagnosis is often found because of other diseases, and the concealment is very high. Fabbro et al. (48) reported an 11-year-old boy who died of distant metastasis of the malignant AS due to renal hematoma due to trauma. Malignant nerve sheath tumor is characterized by rapid growth, usually invading the surrounding tissue structure, early necrosis, and hemorrhagic changes. A malignant tumor is closely related to the increase in tumor diameter (49). As in the case we reported next, it was found by accident during an autopsy. We searched the hospital database and found a 14-year-old boy who was accidentally diagnosed with malignant AS by autopsy. The patient's liver ruptured due to a car accident and he died of excessive blood loss before being sent to the hospital. The adrenal tumor was found during the autopsy requested by the patient's family, and the final pathological diagnosis was malignant schwannoma. Because we only have the pathological results of the patient and a lack of detailed clinical and imaging data, the patient was not included in our case group. Malignant AS is rare than benign AS. We will share the pathological results of this case with the majority of researchers to improve the understanding of the disease. The tumor cells, in this case, showed no capsule under the microscope, mainly in the Antoni A area, with characteristic changes such as verocay body, calcification, necrosis, microcapsules, hyalinization, myxoid stroma, and so on. Immunohistochemical results revealed that S-100 and Vim were positive, CK, EMA, CD34, Des, HMB45, MyoD1, and Melan A were all negative, and the number of Ki-67-positive cells was about 3%. The pathological features of this case of malignant schwannoma are shown in Figure 3. Adrenal malignant peripheral nerve sheath has a high degree of malignancy; metastasis usually occurs within 2 years of discovery, with distant metastasis to the subcutaneous, lung, liver, bone, and other sites common, but lymph node metastasis rare. Because the tumor often grows along the nerve sheath, the 5-year survival rate of malignant peripheral schwannoma patients with neurofibromatosis is about 20%, while the 5-year survival rate of patients without neurofibromatosis is about 50%. The independent risk factors affecting malignant peripheral schwannoma may be related to the history of radiotherapy, positive tumor margin, and Ki-67 index. When Ki-67 > 20%, tumor cells proliferate actively, which often indicates a poor prognosis. At present, there is a lack of unified treatment for malignant peripheral schwannoma. Complete tumor resection combined with postoperative adjuvant radiotherapy is recognized by most scholars, but the therapeutic effect is uncertain, and more clinical data is needed to confirm it.

Our research also has some limitations. First, because of the rarity of this case, this study only included eight cases of AS in a single center for the retrospective study. Larger studies are needed to guide the diagnosis and treatment of AS in the future. Second, due to the long interval before and after diagnosis in eight patients with AS and due to the rapid development of imaging technology in recent years, there is a lack of consistent imaging parameters. Third, all our cases are benign AS, although there is one case of malignant AS found by autopsy, but due to the lack of imaging data, we did not include it in our study, so we only show the imaging features of benign AS. There is a lack of imaging comparison between benign AS and malignant AS. The advantages of our study are as follows: First, up to now, the total number of AS cases reported in the literature is less than 200. Most of the AS in the literature appears in the public view in the form of case reports, and most of the previous literature reports only have imaging data of AS or only pathological data. Therefore, we summarized the clinical data, imaging features, and pathological features of eight cases of AS in order to improve clinicians’ understanding of the disease. Second, because preoperative imaging of AS is difficult to distinguish from other primary adrenal tumors and the correct diagnosis before the operation is particularly important for the treatment of AS, we summarize the diagnosis and differential diagnosis of AS combined with our cases and search for the latest literature. The purpose is to help doctors improve the preoperative diagnosis rate of the disease and reduce the occurrence of overtreatment of patients. Third, we have had 8 cases of AS in our single center in the last 15 years, but fewer than 200 cases of AS have been reported all over the world. The incidence of AS in our medical center is relatively high, and the prognosis of the patients is very good, so we share our treatment experience with the majority of doctors.

## Conclusion

To sum up, it is extremely difficult to diagnose AS before operation, and it is easy to be confused with other adrenal tumors. AS lacks specific clinical and imaging features, but correct diagnosis before the pathological examination is very important for clinical management and surgical decision. When imaging examination indicates a slow-growing retroperitoneal mass, schwannoma should be considered. If the tumor is gradually enlarging or clinical symptoms appear, complete surgical resection should be performed. Pathology is the gold standard of diagnosis. When patients cannot be treated surgically and malignant tumors cannot be excluded, the diagnosis can be confirmed by a puncture biopsy to guide the treatment. Most of the AS are benign tumors, which are mainly resected by radical operation, and the prognosis is good, but there is the possibility of recurrence, and they should be closely monitored.

## Data Availability

The original contributions presented in the study are included in the article/Supplementary Material; further inquiries can be directed to the corresponding author/s.
